# Long-Term Observations of Thickness Changes of Each Retinal Layer following Macular Hole Surgery

**DOI:** 10.1155/2021/4624164

**Published:** 2021-10-19

**Authors:** Atsushi Tada, Shigeki Machida, Yuji Hara, Satoshi Ebihara, Masahiko Ishizuka, Mana Gonmori

**Affiliations:** Department of Ophthalmology, Dokkyo Medical University, Saitama Medical Center, 2-1-50 Minamikoshiagya, Koshigaya, Saitama 343-8555, Japan

## Abstract

**Purpose:**

To determine the long-term changes of the thickness of each retinal layer following macular hole (MH) surgery combined with internal limiting membrane (ILM) peeling.

**Method:**

The medical records of 42 eyes of 42 patients (41 to 86 years of age) who underwent MH surgery with ILM peeling between February 2016 and October 2018 were reviewed. A single surgeon operated on all patients, and all were followed for at least 24 months postoperatively. Spectral-domain optical coherence tomography (OCT) was performed to obtain retinal thickness maps of the parafoveal region corresponding approximately to the ILM peeled area. Each retinal layer was automatically segmented by the embedded software, and thickness maps were constructed for the total retinal layer (TRL), inner RL (IRL), middle RL (MRL), and outer RL (ORL). The averaged value of each retinal layer thickness was analyzed in the temporal/upper, temporal/lower, nasal/upper, and nasal lower quadrants.

**Results:**

The TRL thickness was significantly decreased in the temporal areas postoperatively. The IRL thickness thinned progressively and significantly until 6 months without further thinning in the temporal quadrants. The MRL thickness of all areas was significantly thicker than the baseline values at 0.5 months and then gradually decreased in the temporal regions. However, the thickening in the nasal regions returned to the baseline values after 1.5 months. The ORL decreased transiently relative to the baseline values at 0.5 months in all areas.

**Conclusions:**

The ILM peeling does not affect only the thickness of the inner retina but also the middle and outer retinae in the parafoveal region. The chronological changes of the thickness after surgeries varied among the retinal layers and macular regions.

## 1. Introduction

A macular hole (MH) is caused by vitreoretinal traction that results from a perifoveal posterior vitreous detachment [1–6]. This traction causes a disruption or loss of the “Müller cell cone” in the foveola that precedes the development of a full-thickness MH [6, 7]. Surgical repair of an MH can be achieved by vitrectomy and gas tamponade that leads to an improvement of the visual function [8, 9].

To improve the anatomical success and decrease the recurrence rates, Brooks et al. advocated peeling the internal limiting membrane (ILM) around the MH [10, 11]. Several vital dyes and drugs including indocyanine green (ICG) [12, 13], brilliant blue green (BBG) [14, 15], and trimacinolone acetonide (TA) [16–18] have been used to make the ILM more visible to facilitate the surgical grasping of the ILM. At present, ILM peeling during MH surgery has become the gold standard surgical method for closing an MH.

A large body of evidence has accumulated to show alterations of the retinal structure after ILM peeling during the MH surgery. Initially, Tadayoni et al. reported that a dissociation of the optic nerve fiber layer developed after ILM peeling, and it could affect the visual function [19–22]. Second, a thinning of the temporal retina and thickening of the nasal retina have been reported to occur after ILM peeling by using optical coherence tomography (OCT) [23–25]. The thinning of the temporal retina has been reported to occur predominately in the inner retina as detected in the ganglion-cell complex (GCC) map [26–28]. Third, a retinal displacement toward the optic nerve head after ILM peeling has been shown by tracking the retinal vessels before and after the surgery [29–32].

Spectral-domain OCT (SD-OCT) allows a layer-by-layer analysis of the structure of the retina using thickness maps because high-resolution retinal images can be obtained quickly. Ohta et al. [25] and Faria et al. [33] used retinal thickness maps to demonstrate a thinning of the inner retina and a thickening of the outer retina at certain postoperative times. These findings indicated that the structural changes were not confined to the inner retina where the surgical procedures were performed. However, a long-term study of the chronological changes of thickness of the different retinal layers after MH surgery has not been done.

Thus, the purpose of this study was to determine long-term changes of the thickness of each retinal layer using the thickness map obtained by SD-OCT following MH surgery combined with ILM peeling. We shall show that the ILM peeling affected not only the inner retina but also the middle and outer retinal layers in the parafoveal region. In addition, we also found that the changes varied with the postoperative time.

## 2. Methods

### 2.1. Study Patients

Forty-two eyes of 42 patients underwent the MH surgery combined with ILM peeling between February 2016 and September 2018 in the Dokkyo Medical University Saitama Medical Center. There were 22 women and 20 men whose age ranged from 41 to 86 years with a mean of 67.6 ± 7.3 years (average ± standard deviation). The patients were examined preoperatively and at 0.5, 3, 6, 9, 12, 18, and 24 months postoperatively, and their medical records were reviewed. We included patients whose MHs had been confirmed to be closed by SD-OCT at 0.5 months after the surgery. All patients underwent comprehensive ophthalmological examinations including measurements of the best-corrected visual acuity (BCVA) and slit-lamp biomicroscopic and indirect ophthalmoscopic examinations with dilated pupils. The MHs were classified into stages according to the SD-OCT findings: Stage 1 (*n* = 2), Stage 2 (*n* = 17), Stage 3 (*n* = 15), and Stage 4 (*n* = 8) [1–6]. The base diameter of the MHs ranged from 105 to 1371 *μ*m (average ± standard deviation dimension 599 ± 307 *μ*m) and the minimum diameter from 70 to 641 *μ*m (263 ± 149 *μ*m). These diameters were obtained from the vertical and horizontal sections through the center of the MHs and were averaged.

The exclusion criteria included macular pathologies other than MH, retinal vascular diseases, history of other ocular disorders including uveitis, severe dry eye, trauma and glaucoma, and systemic disorders that could affect the retina such as diabetes, uncontrolled hypertension, inflammatory bowel disease, and prior ocular surgery.

This research was approved by the Institutional Review Board of Dokkyo Medical University and conducted in accordance with the Institutional Guidelines, and the procedures conformed to the tenets of the Declaration of Helsinki.

### 2.2. Surgical Procedures

Standard 3-port, 25-gauge pars plana vitrectomy combined with phacoemulsification and aspiration with an implantation of an intraocular lens (NX-70, Advanced Vision Science, Inc., Coleta, CA, USA) was performed on all patients using the Constellation Vision SystemⓇ (Alcon Inc., Geneva, Switzerland). Preservative-free triamcinolone acetonide (TA; MaQaid, Wakamoto Pharmaceutical Co., Ltd, Tokyo, Japan) was suspended in 4 ml balanced salt solution (BSS plus, Alcon Japan, Tokyo, Japan) and injected intravitreally during vitrectomy to make the posterior hyaloid membrane more visible. BBG was dissolved in BSS plus to a concentration of 0.025%, and approximately 0.2 ml of the dye solution was injected intravitreally with a gentle stream directed toward the posterior pole of the eye. The dye was removed from the vitreous cavity by infusion and aspiration.

Then, the ILM was grasped with an ILM forceps, and the ILM was peeled around the macular hole with a size of approximately 3-disc diameters. Air-fluid exchange was performed followed by an injection of 20% sulfur hexafluoride (SF_6_). All surgical procedures were performed by one of the authors (SM). All patients were instructed to maintain a face-down position for at least 2 days after the surgery.

### 2.3. Optical Coherence Tomography (OCT)

**Figure 1:**
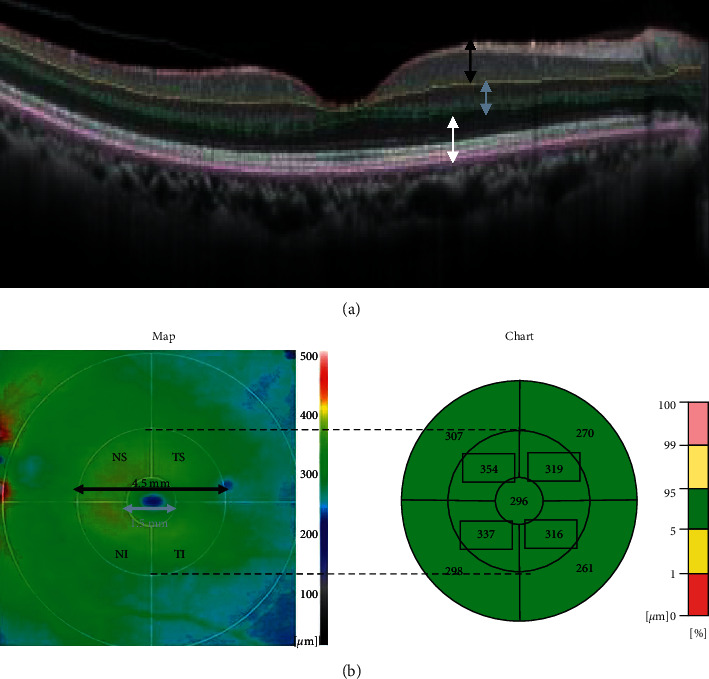
Cross-sectional images obtained by SD-OCT showing how measurements of the retinal thickness of the inner (black arrow), middle (gray arrow), and outer retinal layers (white arrow) were made. (a) Retinal thickness map of the inner retinal layer. (b) Inner and middle circles of 1.5 and 4.5 mm diameters were placed on the center of the fovea. Averaged thicknesses for the temporal/superior (TS), temporal/inferior (TI), nasal/superior (NS), and nasal/inferior (NI) quadrants of the middle circle are presented in the thickness chart (encompassed by squares). SD-OCT, spectral-domain optical coherence tomography.

OCT scan images were acquired with a spectral-domain OCT instrument (SD-OCT, RS-3000 Advance, Nidek Co. LTD., Gamagori, Aichi, Japan; Figure 1(a)). The retinal thickness was measured at 512 × 128 points in the posterior pole of the eye (Figure 1(b)). The tracking system of the OCT system reduced the effects of eye movements that allowed us to measure the thickness at each retinal point reliably.

We used the thickness charts for the analyses. The mean thickness was determined for each quarter of an annulus with an inner diameter of 1.5 mm and an outer diameter of 4.5 mm. The selected areas were marked out in squares (Figure 1(b)). The quarter annuli were designated as the temporal/superior (TS), temporal/inferior (TI), nasal/superior (NS), and nasal/inferior (NI) quadrants.

All thickness measurements were made automatically by the autosegmentation software embedded in the OCT device. The total retinal layer (TRL) from the ILM to the retinal pigment epithelium (RPE), the inner retinal layer (IRL) from the ILM to the inner plexiform layer (black arrows in Figure 1(a)), the middle retinal layer (MRL) from the inner nuclear layer to the outer plexiform layer (gray arrows in Figure 1(a)), and the outer retinal layer (ORL) from the outer nuclear layer to RPE (white arrows in Figure 1(a)) were measured to obtain the thickness map for each retinal layer. Manual corrections were made when segmentation errors were found.

### 2.4. Statistical Analyses

The GraphPad Prism version 8.1.2 (332) (GraphPad software, San Diego, CA, USA) software was used for statistical analyses. The measured data are presented as the means ± standard deviations (SDs). The distribution of the measured data was examined by the Kolmogorov–Smirnov test. The statistical significance of the differences for diseased eyes at 0.5, 3, 6, 9, 12, 18, and 24 months following the surgery was evaluated by the repeated-measures one-way ANOVA tests. In addition, the Turkey's multiple comparison tests were performed after the ANOVA as post hoc tests. Bartlett's test was used to correct the *P* values. A statistical level of significance was accepted at *P* < 0.05.

## 3. Results

### 3.1. Representative Case

**Figure 2:**
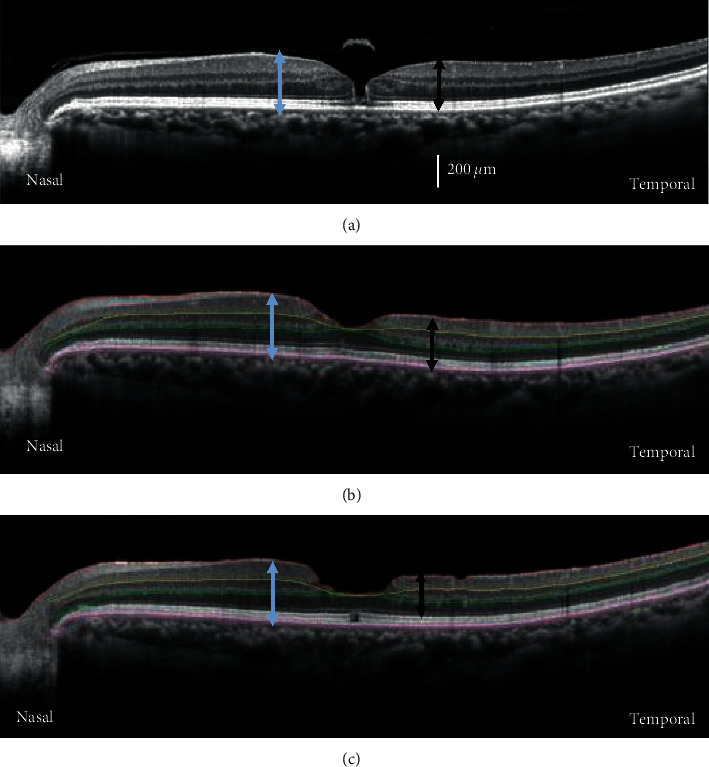
Representative cross-sectional SD-OCT images obtained by horizontal scans from eyes with a MH preoperatively (baseline, (a)) and postoperatively at the 0.5 (b) and 24 (c) months. The arrows placed at 1.0 mm from the fovea indicate the total retinal thickness in the nasal (gray arrows) and temporal (black arrows) retina. SD-OCT, spectral-domain optical coherence tomography; MH, macular hole.

B-scan SD-OCT images from an eye with an MH at the preoperative baseline, 0.5, and 24 months postoperatively are shown in Figure 2. The thickness of the parafoveal nasal retina appears to be unchanged (gray arrows) relative to that of the baseline image (Figure 2(a)) until 24 months (Figure 2(c)). However, the temporal retina was thinner than the baseline at 0.5 (Figure 2(b)) and 24 months (black arrows). The arrows were placed at 1.0 mm from the fovea.

### 3.2. Mean Thickness of Total Retinal Layer (TRL)

**Figure 3:**
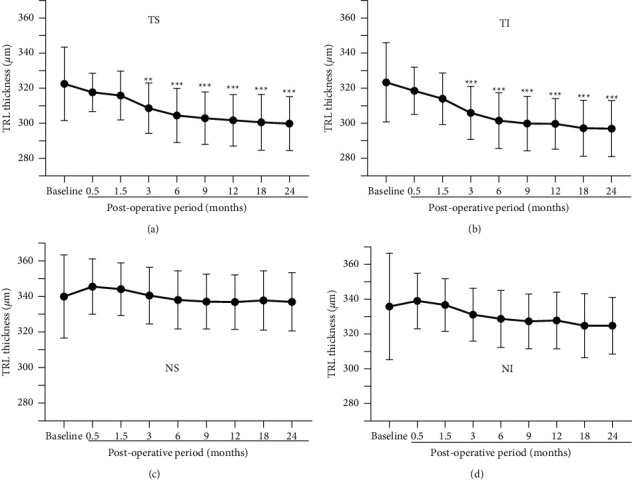
Mean thicknesses of the total retinal layer (TRL) are plotted for the MH eyes that had undergone vitrectomy with ILM peeling. In the temporal/superior (a) and temporal/inferior (b) quadrants, the TRL thickness decreases significantly with postoperative time. A significant thinning of the TRL was found between the baseline (preoperative eyes) and postoperative eyes at 3 months and thereafter. MH, macular hole; ILM, internal limiting membrane; TS, temporal/superior; TI, temporal/inferior; NS, nasal/superior; NI, nasal/inferior. Asterisks represent significant differences between the control and operated eyes.  ^*∗∗*^*P* < 0.005,  ^*∗∗∗*^*P* < 0.0001.

The mean thickness of the TRL is plotted against the postoperative times for each quadrant to assess the chronological changes (Figure 3). In the temporal quadrants (Figures 3(a) and 3(b)), the TRL thickness gradually and significantly decreased with time (*P* < 0.0001) with a significant reduction compared to that of the baseline at 3, 6, 9, 12, 18, and 24 months (*P* < 0.05 to 0.0001). In the nasal quadrants (Figures 3(c) and 3(d)), the TRL thicknesses did not change significantly with time.

### 3.3. Mean Thicknesses of Inner Retinal Layer (IRL)

**Figure 4:**
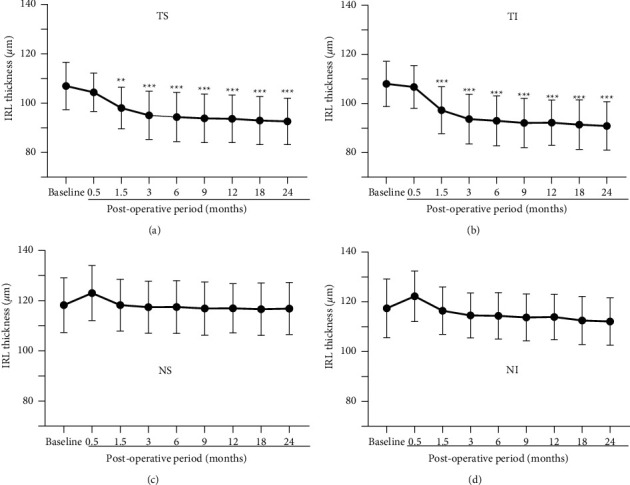
Mean thicknesses of the inner retinal layer (IRL) are plotted for the MH eyes treated with ILM peeling. In the temporal/superior (a) and temporal/inferior (b) quadrants, the IRL thickness significantly decreases with postoperative time up to 6 months with subsequent stability. Significant thinning of the IRL was found between the baseline (preoperative eyes) and postoperative eyes at 1.5 months and thereafter. MH, macular hole; ILM, internal limiting membrane; TS, temporal/superior; TI, temporal/inferior; NS, nasal/superior; NI, nasal/inferior. Asterisks represent significant differences between the control and operated eyes.  ^*∗∗*^*P* < 0.005;  ^*∗∗∗*^*P* < 0.0001.

The mean thicknesses of the IRL are plotted against the postoperative times for each quadrant to assess chronological changes (Figure 4). In the temporal quadrants (Figures 4(a) and 4(b)), the IRL thickness was not significantly different from the baseline values at 0.5 months, and it progressively decreased until 6 months and then remained unchanged throughout the remaining study period (*P* < 0.0001). The IRL was significantly thinner after 1.5 months than that of the baseline (*P* < 0.005 − 0.0001). In the nasal quadrants, the IRL thickness was slightly increased and then decreased postoperatively, but the difference from that of the baseline was not significant.

### 3.4. Mean Thickness of Middle Retinal Layer (MRL)

**Figure 5:**
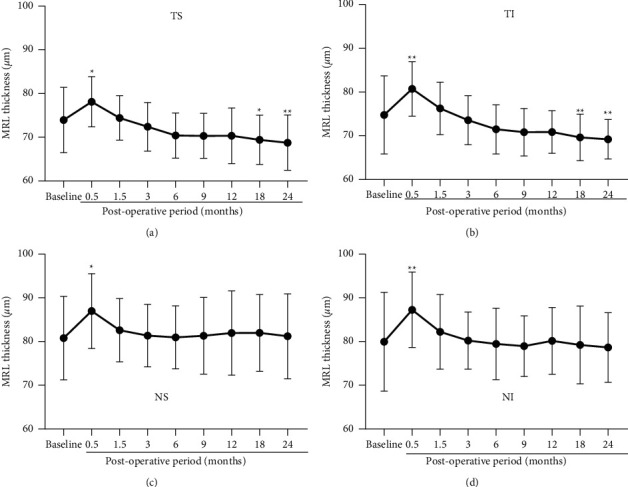
Mean thicknesses of the middle retinal layer (MRL) are plotted for the MH eyes treated with ILM peeling. In the temporal/superior (a), temporal/inferior (b), nasal/superior (c), and nasal/inferior quadrants (d), the MRL of the treated eyes is thicker at 0.5 months with significant difference from that of the baseline (preoperative eyes). In the temporal quadrants, the MRL is thinner with significant difference from that of the baseline after the month 18. MH, macular hole; ILM, internal limiting membrane; TS, temporal/superior; TI, temporal/inferior; NS, nasal/superior; NI, nasal/inferior. Asterisks represent significant differences between the control and operated eyes.  ^*∗*^*P* < 0.05,  ^*∗∗*^*P* < 0.005.

The mean thicknesses of the MRL are plotted against the postoperative time for each quadrant (Figure 5). In the temporal quadrants (Figures 5(a) and 5(b)), the MRL was significantly thicker than that of the baseline values at 0.5 months (*P* < 0.05 − 0.005). Then it gradually decreased with a significant reduction at 18 and 24 months compared to that of the baseline (*P* < 0.05 − 0.005.

In the nasal quadrants (Figures 5(c) and 5(d)), there was a significant increase of the MRL thickness at 0.5 months (*P* < 0.05 − 0.005). Thereafter, the MRL thickness decreased for 1.5 months without a sequential thinning.

### 3.5. Mean Thickness of Outer Retinal Layer (ORL)

**Figure 6:**
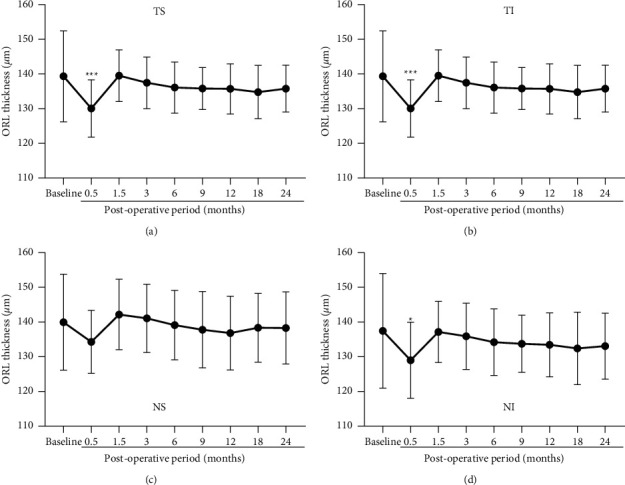
Mean thicknesses of the outer retinal layer (ORL) are plotted for the MH eyes treated with ILM peeling. In the temporal/superior (a), temporal/inferior (b), nasal/superior (c), and nasal/inferior quadrants (d), the ORL thickness of the treated eyes decreased at the month 0.5 with significant difference from that of the baseline (preoperative eyes) in all quadrants except for the NS. MH, macular hole; ILM, internal limiting membrane; TS, temporal/superior; TI, temporal/inferior; NS, nasal/superior; NI, nasal/inferior. Asterisks represent significant differences between the control and operated eyes.  ^*∗*^*P* < 0.05,  ^*∗∗*^*P* < 0.0001.

The mean ORL thicknesses are plotted against the postoperative times for each quadrant to assess chronological changes (Figure 6). There was a significant decrease of the ORL thickness that peaked at 0.5 months in all quadrants except for the NS quadrant (*P* < 0.05 for NI; *P* < 0.0001 for TS and TI) followed by a quick return to the baseline values. The ONL thicknesses were slightly thicker than the baseline values at 1.5 months without statistical significance.

To investigate how MH sizes affected the changes of the retinal thicknesses postoperatively, we conducted same analysis after classifying the patients into two groups: the large MH group (>400 *μ*m minimum diameter) and the small MH group (≦400 *μ*m). We could not find any significant differences in the thickness changes in the TRL, IRL, MRL, and ORL between the two groups.

## 4. Discussion

To the best of our knowledge, the present study is the first to determine the sequential and long-term changes in thickness of each retinal layer using thickness maps of the macular area following MH closure with ILM peeling. The results demonstrated that the thickness of each retinal layer changed with increasing postoperative times.

### 4.1. Thinning of Temporal Retina

Baba et al. reported a thinning of the GCC after MH surgery and showed that the thinning progressed until 6 months postoperatively [26]. Our long-term observations demonstrated that the temporal IRL thickness progressively decreased until 6 months and then remained stable for 24 months. Because a progressive thinning was also seen in the MRL for at least 6 months, the thinning of the TRL in the temporal retina was due to a thinning of both the IRL and MRL. Because the ILM is the basement membrane of the Müller cells, Spaide suggested that the trauma and healing process of the Müller cells may result in a dimpling of the inner retina [28]. However, this cannot be the sole reason why the retinal thinning takes place predominantly in the temporal retina in spite of the peeling of the ILM completely around the fovea. There may be other factors that contribute to the thinning of the temporal retina.

Recent studies have tracked the postoperative movements of the retinal vessels, and the results showed that the retina was displaced toward the optic nerve head after ILM peeling [29–32]. In addition, there are also reports that the fovea is displaced toward the optic disc after ILM peeling [31, 32], which suggests that not only the IRL but also the ORL and MRL are displaced toward the optic nerve head.

The ILM plays an important role in maintaining the rigidity of the retina [34, 35]. After ILM peeling, the loss of the retinal rigidity is supposed to contribute to the shrinkage of the retinal nerve fibers resulting in the retinal displacement toward the optic nerve head. Because the displacement toward the optic nerve head is greater for the temporal retina than the nasal retina [29, 32], there should be a displacement of the retinal cells toward the optic nerve head possibly resulting in the thinning of the temporal retina. Imamura and Ishida have demonstrated that the size of the MH was correlated with the temporal retinal thinning, and they suggested that the retinal movement toward the optic disc contributed to the thinning of the temporal retina [36]. It would be interesting to investigate the relationship between retina movement and changes of the retinal thickness in more detail even though we could not find any significant differences in the thickness changes of each retinal layer between the large and small MH groups.

### 4.2. Transient Increase and Decrease of Retinal Thicknesses

A transient increase of the retinal thickness relative to the baseline values was found for the MRL at the early postoperative times that corresponded to the transient thinning of the ORL at 0.5 months. It has been reported that it takes at least 12 months for the foveal microstructures to be restored following MH surgery [37]. Therefore, the restoration cannot explain the transient changes of the ORL and MRL at the earlier postoperative periods. The ILM is the basement membrane of the Müller cells that extend from the inner to the outer retina. Therefore, physical stress induced by surgical procedures such as ILM peeling could influence the Müller cell throughout all retinal layers possibly causing inflammation and gliosis. These pathological changes may contribute to the transient changes of the retinal thickness of all retinal layers.

### 4.3. Differences from Previous Studies

The results of earlier studies demonstrated that the outer retina thickened at 6 months after ILM peeling in the parafoveal and perifoveal regions in the temporal retina [25, 33]. In contrast, our results showed a transient decrease of the ORL thickness at 0.5 months followed by a slight nonsignificant increase at 1.5 months, and it returned to the baseline level thereafter. In earlier studies, the transient and slight increase of the ORL thickness may have been detected. Alternatively, in these studies, the borders of the outer nuclear layer thickness may have been different from what we designated the ORL thickness as the thickness from the outer nuclear layer to the RPE.

### 4.4. Limitations of This Study

Several limitations of this study can be raised. First, because this study was conducted at a single center, we were able to study only a small number of patients. However, as surgical devices and surgeons remained unchanged throughout the study, variations in the surgical procedures were minimal. Second, the retrospective nature of the study may have weakened the strength of the evidence although the patients were followed periodically. Third, the retinal area of the ILM peeling was different among the patients, while the diameter of the ILM peeled area was intended to be 3-disc diameters. Finally, we did not evaluate the retinal function along with retinal morphology. It would have been interesting to determine the functional changes using the focal macular electoretinography (fmERG) and determine the significance of the correlations between the retinal thickness of each retinal layer and the fmERG. This is especially important because the fmERGs can assess the function of the macular region in a layer-by-layer method [38–40].

## 5. Conclusions

Long-term longitudinal observations can show the changes in the thickness of each retinal layer postoperatively. The layer-by-layer analysis using OCT-determined thickness maps demonstrated that the IRL and MRL thinning contributes to the total thickness reduction in the temporal parafoveal region following ILM peeling. Transient changes of the MRL and ORL thicknesses were seen as early as 2 weeks postoperatively. The ILM peeling affects not only the inner retina but also the middle and outer retinae in the parafoveal region.

## Data Availability

The data used to support the findings of this study are available from the corresponding author upon request.
